# Individual Characteristics Associated with Mismatches between Self-Reported and Accelerometer-Measured Physical Activity

**DOI:** 10.1371/journal.pone.0099636

**Published:** 2014-06-11

**Authors:** Mark A. Tully, Jenna Panter, David Ogilvie

**Affiliations:** 1 UKCRC Centre of Excellence for Public Health (NI), Centre for Public Health, Institute for Clinical Science (B), Royal Victoria Hospital, Belfast, United Kingdom; 2 MRC Epidemiology Unit and UKCRC Centre for Diet and Activity Research (CEDAR), University of Cambridge School of Clinical Medicine, Cambridge Biomedical Campus, Cambridge, United Kingdom; Universidad Pablo de Olavide, Centro Andaluz de Biología del Desarrollo-CSIC, Spain

## Abstract

**Background:**

Accurate assessment tools are required for the surveillance of physical activity (PA) levels and the assessment of the effect of interventions. In addition, increasing awareness of PA is often used as the first step in pragmatic behavioural interventions, as discrepancies between the amount of activity an individual perceives they do and the amount actually undertaken may act as a barrier to change. Previous research has demonstrated differences in the amount of activity individuals report doing, compared to their level of physical activity when measured with an accelerometer. Understanding the characteristics of those whose PA level is ranked differently when measured with either self-report or accelerometry is important as it may inform the choice of instrument for future research. The aim of this project was to determine which individual characteristics are associated with differences between self-reported and accelerometer measured physical activity.

**Methods:**

Participant data from the 2009 wave of the Commuting and Health in Cambridge study were used. Quartiles of self-reported and accelerometer-measured PA were derived by ranking each measure from lowest to highest. These quartiles were compared to determine whether individuals’ physical activity was ranked higher by either method. Multinomial logistic regression models were used to investigate the individual characteristics associated with different categories of mismatch.

**Results:**

Data from 486 participants (70% female) were included in the analysis. In adjusted analyses, the physical activity of overweight or obese individuals was significantly more likely to be ranked higher by self-report than by accelerometer than that of normal-weight individuals (OR = 2.07, 95%CI = 1.28–3.34), particularly among women (OR = 3.97, 95%CI = 2.11–7.47).

**Conclusions:**

There was a greater likelihood of mismatch between self-reported and accelerometer measured physical activity levels in overweight or obese adults. Future studies in overweight or obese adults should consider employing both methods of measurement.

## Introduction

Physical inactivity is the fourth leading cause of death and disease worldwide [1] and there have been considerable efforts to address declining levels of physical activity (PA). For the surveillance of PA levels and the assessment of the effect of interventions, accurate assessment tools are required [2], [3] as imprecision may affect the apparent magnitude of any changes [4]. In addition, increasing awareness of PA is often used as the first step in pragmatic behavioural interventions, as discrepancies between the amount of activity an individual perceives they do and the amount actually undertaken may act as a barrier to change [5]. Understanding the characteristics of those who misperceive their activity levels may be important as such discrepancies may moderate the effects of interventions.

Numerous methods exist for measuring PA, ranging from extremely brief self-report questionnaires for use in primary care consultations [6] to more complex instruments such as accelerometers [7]. Self-report methods are often preferred over accelerometers in surveillance as they are cheap to administer, take little time to complete, require limited technical expertise in analysis, can offer information on the context of activities and record more than just ambulatory activities [8]. On the other hand, accelerometers provide more accurate and detailed measurement of PA, may be used to provide feedback to the participant on their progress in meeting goals for changing behaviour, and are not prone to certain biases in self-report methods such as recall and social desirability bias [9]. Such inaccuracies may result in differences between self-reported and accelerometer-measured PA and explain the weak-to-moderate correlations observed between these [3].

Using a large nationally representative US dataset, Tucker et al [10] have demonstrated that 60% of individuals were classified as meeting current guidelines for PA using self-reported measures compared to 9% using accelerometers. The authors concluded that this suggested a significant overestimation of PA using self-report. Stratified analysis of the relationship between self-reported and accelerometer-measured PA has indicated stronger correlation in men [3], suggesting that it may be possible to identify groups for whom self-report is more or less valid.

Previous research has examined factors associated with discrepancies between a person’s general perception of their activity level (e.g. low, moderate or highly active) and the amount of activity as measured by an accelerometer. Adults who rate themselves as more active than indicated by accelerometry have been found to be more likely to have a higher body mass index (BMI) [11], [12] or level of education [12] and to perceive themselves as healthy [12], [13], and less likely to report an intention to change their PA [13]. In contrast, there is limited evidence of which individual (e.g. health or socio-demographic) characteristics are associated with mismatches between more detailed, validated self-reported measures of PA and those derived from objective measures. To our knowledge, only one previous study has sought to determine correlates with mismatches in reporting PA with a validated self-report instrument. This study compared the Baecke PA Questionnaire with energy expenditure (EE) using doubly-labeled water [14] and demonstrated that total EE was more likely to be over-reported in overweight women than in those of normal weight. This study was conducted in only 75 women and involved an objective measure that is infrequently used in free-living conditions. Further research is therefore required to confirm previous findings in larger, more generalisable populations using more commonly used objective measures.

Understanding the characteristics of those whose PA level is ranked differently when measured with either self-report or accelerometry is important as it may inform the choice of instrument for future research. The aim of this study was to determine which individual characteristics are associated with mismatches between self-reported and accelerometer-measured PA in a large sample of working adults.

## Methods

### Participants

This paper uses baseline data from the *Commuting and Health in Cambridge* study, of which full details of the protocol have been published previously [15]. In summary, 1164 adults aged 16 and over working in Cambridge and living within 30 km of the city were recruited in 2009, predominantly via workplaces [16]. All participants completed a questionnaire [16] that included questions on recent PA and commuting behaviours. In addition to their date of birth, sex, height and weight, individuals were asked to identify their highest level of educational attainment and housing tenure and the number of cars in their household. A sub-sample of participants were issued with an accelerometer for one week [17], [18]. The study was approved by the Hertfordshire Research Ethics Committee and all participants gave written informed consent.

### Measures of Physical Activity

Self-reported PA was measured using the Recent Physical Activity Questionnaire (RPAQ). RPAQ measures PA in four domains (domestic, travel, work and recreation) over the last four weeks, from which total and PA energy expenditure (PAEE) per week is estimated. RPAQ has been shown to have reasonable validity against doubly-labelled water in ranking EE levels of individuals (r = 0.39) [19]. PAEE was calculated as the sum of EE expenditure in each domain, according to the method described by Besson et al [19].

PA was objectively measured for seven consecutive days using either an Actigraph GT1M or GT3X accelerometer worn over the right hip on an elasticated belt. Actigraph accelerometers are small, lightweight devices that record movement, have been shown to provide reliable measures of activity [20], [21]. Data were stored at 5-second epochs and uniaxial data files were processed using the MAHUFFE Software package (http://www.mrc-epid.cam.ac.uk) to calculate average daily counts per minute (CPM) for each participant. Non-wear time was defined as a run of zero counts lasting more than 20 minutes [22]. At least three valid days were required for inclusion in the analysis, each defined as a 24-hour period in which more than 600 minutes of wear time were recorded.

### Analysis

Participants were included in this analysis if they provided valid self-report and accelerometer data. Outputs from the two instruments were not directly compared because previous research has shown that RPAQ has acceptable validity for ranking individual’s EE for vigorous PA, but lower levels of validity for light or moderate intensity activity [19]. Furthermore, average daily CPM, rather than minutes of moderate and vigorous PA (MVPA), from Actigraph were used in the analysis because we determined that this summary variable would provide a more comparable measure of PA to that derived from the RPAQ. For many analyses, minutes of MVPA provide an appropriate measure to reflect the time spent in health enhancing physical activity (at a moderate intensity level or above) [23]. However, by including the energy expenditure from activities below and above this level, the RPAQ produces a broader measure of *overall* PAEE. For a less biased comparison, therefore, average daily CPM were compared to PAEE from RPAQ.

Quartiles of self-reported and accelerometer-measured PA were derived by ranking each measure from lowest to highest. Participants were assigned to one of three groups according to whether their PA was (i) ranked equally (i.e. categorised in the same quartile) by self-report and accelerometer, (ii) ranked higher (i.e. categorised in a higher quartile) by self-report than by accelerometer, or (iii) ranked higher by accelerometer than by self-report (Table 1).

**Table 1:** Classification of participants into groups by comparing self-reported and accelerometer measured PA.

	Actigraph^b^				
	*Quartile of PA*	*1*	*2*	*3*	*4*
**RPAQ** a	1	**PA ranked equally (n = 43)**	PA ranked higher byaccelerometer (n = 29)	PA ranked higher byaccelerometer (n = 30)	PA ranked higher byaccelerometer (n = 18)
	2	PA ranked higher byself-report (n = 36)	**PA ranked equally (n = 31)**	PA ranked higher byaccelerometer (n = 27)	PA ranked higher byaccelerometer (n = 25)
	3	PA ranked higher byself-report (n = 26)	PA ranked higher byself-report (n = 28)	**PA ranked equally (n = 35)**	PA ranked higher byaccelerometer (n = 31)
	4	PA ranked higher byself-report (n = 14)	PA ranked higher byself-report (n = 31)	PA ranked higher byself-report (n = 28)	**PA ranked equally (n = 46)**

a RPAQ: Physical activity measured using the Recent Physical Activity Questionnaire.

b Actigraph: Physical activity measured using an Actigraph accelerometer.

Data were described using percentages. Multinomial logistic regression models were specified to investigate associations between individual characteristics (age, BMI, highest educational attainment, number of cars, home ownership, cycling to work) and the three categorical outcomes described above. We observed an association between sex and BMI (whereby men had a significantly higher BMI than women, p = 0.02) and therefore stratified our analysis by sex. Variables found to be significant in univariate analysis (p<0.25) were carried forward into a multivariable analysis [24]. Analysis was conducted using SPSS v18.

## Results

### Characteristics of the Sample

Of the 1164 individuals who completed the baseline questionnaire, 714 were also issued with an Actigraph accelerometer, 499 returned a completed questionnaire and accelerometer and 486 provided valid accelerometer data. These participants were approximately 1.5 years older on average, and more likely to have access to a car and to own their home, than the overall study sample (n = 1164) [25]. A further eight individuals did not complete the PA questionnaire in full, resulting in 478 individuals (67% of those issued with both instruments) eligible for inclusion in this analysis. The final sample were a relatively socio-economically advantaged group, most of whom owned their home (80%), had access to at least one car (89%) and were educated to at least degree level (71%) (Table 2).

**Table 2:** Descriptive statistics of participants.

		Full cohort	Males	Females	p^a^
		(n = 478)	(n = 144)	(n = 334)	
Physical Activity	Counts per minute (Mean±SD)	360.81±130.18	389.83±132.23	348.30±127.46	0.001
	PAEE (kJ/d) (Mean±SD)	4805.52±2489.76	6471.43±2797.00	4087.29±1949.99	0.001
Classification of agreement	PA ranked equally by self-report and accelerometer	32 (155)	33 (47)	33 (108)	0.02
	PA ranked higher by accelerometer than self-report	34 (160)	20 (29)	39 (131)	
	PA ranked higher by self-report than accelerometer	34 (163)	47 (68)	28 (95)	
Age Category	20–29 years	14 (66)	8 (11)	17 (55)	0.10
	30–39 years	27 (129)	26 (38)	27 (91)	
	40–49 years	27 (128)	33 (48)	24 (80)	
	50–69 years	32 (155)	33 (47)	32 (108)	
Weight^b^	Not overweight (<25 kg/m^2^)	62 (295)	54 (78)	65 (217)	0.03
	Overweight or obese (≥25 kg/m^2^)	38 (183)	46 (66)	35 (117)	
Home Ownership	Rents	20 (96)	19 (27)	21 (69)	0.62
	Owns	80 (381)	81 (117)	79 (264)	
Number of Cars	No Car	10 (49)	14 (20)	9 (29)	0.49
	1 car	41 (193)	37 (54)	41 (139)	
	2 or more cars	49 (236)	49 (70)	50 (166)	
Highest level of Education^b^	Up to degree level	29 (138)	19 (27)	33 (111)	0.001
	Degree level or higher	71 (338)	81 (117)	67 (221)	
Travel to work by bicycle	Never	54 (258)	41 (59)	60 (199)	0.001
	Occasionally, usually or always	46 (220)	59 (85)	40 (135)	

Values are percentage (number of participants) unless otherwise stated. Data were collected between May and November 2009 in Cambridge, UK.

a differences between males and females assessed using one-way ANOVA for continuously distributed variables and Mann-Whitney U test for non-parametric data.

b n<478 due to missing data.

In the full cohort, the PA of 160 individuals (34% of overall sample; 131 females, 29 males) was ranked higher by accelerometer than by self-report, the PA of 163 individuals (34% of overall sample; 95 females, 68 males) was ranked higher by self-report than by accelerometer, and PA was ranked equally by self-report and accelerometer in 155 individuals (32% of overall sample; 108 females, 47 males). Men were found to be more active than women using both self-report and accelerometry (p = 0.01). A greater proportion of men than women were ranked in a higher quartile of PA by self-report than by accelerometer (47% vs. 28%), and a greater proportion of women than men were ranked in a higher quartile of PA by accelerometer than by self-report (39% vs. 20%) (both p = 0.02) (Table 3).

**Table 3:** Descriptive characteristics of participants by the comparison of ranking of PA by self-report and an accelerometer.

		Full Cohort (n = 478)			Males (n = 144)			Females (n = 334)		
		PA rankedequally byself-reportandaccelerometer	PA rankedhigher byaccelerometerthanself-report	PA rankedhigher byself-reportthanaccelerometer	PA rankedequally byself-reportand accelerometer	PA rankedhigher byaccelerometer thanself-report	PA rankedhigher byself-reportthanaccelerometer	PA rankedequally byself-reportandaccelerometer	PA rankedhigher byaccelerometerthanself-report	PA rankedhigher byself-reportthanaccelerometer
		(n = 155)	(n = 160)	(n = 163)	(n = 47)	(n = 29)	(n = 68)	(n = 108)	(n = 131)	(n = 95)
Physical Activity	CPM	372.84	433.74	277.79	466.14	468.82	303.41	332.24	425.98	259.45
	*Mean*±*SD*	±141.29	±119.57	±67.55	±120.13	±141.58	±68.33	±130.54	±113.28	±61.01
	PAEE	5041.49	3282.34	6076.28	7356.06	3669.29	7055.03	4034.23	3196.68	5375.71
	*Mean*±*SD*	±2816.58	±117.92	±2320.39	±2822.46	±1236.14	±2509.18	±2148.44	±1149.49	±1898.04
Sex	Male	30.3 (47)	18.1 (29)	41.7 (68)	-	-	-	-	-	-
	Female	69.7 (108)	81.9 (131)	58.3 (95)	-	-	-	-	-	-
Age	20–29 yrs	11.6 (18)	18.1 (29)	11.7 (19)	36.4 (4)	27.3 (3)	36.4 (4)	25.5 (14)	47.3 (26)	27.3 (15)
	30–39 yrs	29.0 (45)	25.6 (41)	26.4 (43)	28.9 (11)	18.4 (7)	52.6 (20)	37.4 (34)	37.4 (34)	25.3 (23)
	40–49 yrs	28.4 (44)	23.1 (37)	28.8 (47)	37.5 (18)	10.4 (5)	52.1 (25)	32.5 (26)	40.0 (32)	27.5 (22)
	50–69 yrs	31.0 (48)	33.1 (53)	33.1 (54)	29.8 (14)	29.8 (14)	40.4 (19)	31.5 (34)	36.1 (39)	32.4 (35)
BMI	Not overweight (<25 kg/m^2^)	63.9 (99)	75.0 (120)	46.6 (76)	28.2 (22)	24.4 (19)	47.4 (37)	35.5 (77)	46.5 (101)	18.0 (39)
	Overweight or obese (≥25 kg/m^2^)	36.1 (56)	25.0 (40)	53.4 (87)	37.9 (25)	15.2 (10)	47.0 (31)	26.5 (31)	25.6 (30)	47.9 (56)
Home Ownership	Rents	18.7 (29)	21.9 (35)	19.8 (32)	33.3 (9)	22.2 (6)	44.4 (12)	29.0 (20)	42.0 (29)	29.0 (20)
	Owns	81.3 (126)	78.1 (125)	80.2 (130)	32.5 (38)	19.7 (23)	47.9 (56)	33.3 (88)	38.6 (102)	28.0 (74)
Number of Cars	No Car	13 (8.4)	13.8 (22)	8.6 (14)	25.0 (5)	40.0 (8)	35.0 (7)	27.6 (8)	48.3 (14)	24.1 (7)
	1 car	41.9 (65)	39.4 (63)	39.9 (65)	37.0 (20)	20.4 (11)	42.6 (23)	32.4 (45)	37.4 (52)	30.2 (42)
	2 or more cars	49.7 (77)	46.9 (75)	51.5 (84)	31.4 (22)	14.3 (10)	54.3 (38)	33.1 (55)	39.2 (65)	27.7 (46)
Highest educational qualification	Up to degree level	24.5 (38)	34.4 (55)	27.8 (45)	22.2 (6)	14.8 (4)	63.0 (17)	28.8 (32)	45.9 (51)	25.2 (28)
	Degree level or higher	74.8 (116)	65.6 (105)	72.2 (117)	35.0 (41)	21.4 (25)	43.6 (51)	33.9 (75)	36.2 (80)	29.9 (66)
Travel to work by bicycle	Never	52.9 (82)	61.9 (99)	47.2 (77)	35.6 (21)	22.0 (13)	42.4 (25)	30.7 (61)	43.2 (86)	26.1 (52)
	Occasionally, usually or always	47.1 (73)	38.1 (61)	52.8 (86)	30.6 (26)	18.8 (16)	50.6 (43)	34.8 (47)	33.3 (45)	31.9 (43)

Values are percentage (number of participants) unless otherwise stated. Data were collected between May and November 2009 in Cambridge, UK.

### Univariable Associations

In the full sample, univariable analysis indicated that individuals whose PA was ranked higher by accelerometer than by self-report were more likely to be female, aged 20–29 years, not overweight or obese, living in a household without a car, not to be educated to degree level and not to cycle to work (p<0.25) (Table 4). Conversely, those whose PA was ranked higher by self-report than by accelerometer were more likely to be male and overweight or obese (both p<0.25) (Table 4).

**Table 4:** Associations between individual characteristics and the ranking of physical activity by self-report and an accelerometer in the full cohort.

		*Univariate Analysis*			*Multivariable Analysis*		
		OR	95% CI	p	OR	95% CI	p
**PA ranked higher by accelerometer than self-report (n = 160)**							
Sex (reference = female)^a^	Male	0.50	0.30–0.86	0.12	0.57	0.33–1.00	0.05
Age (reference = 20–29 yrs)^a^	30–39 yrs	0.57	0.28–1.17	0.12	0.69	0.33–1.45	0.32
	40–49 yrs	0.52	0.25–1.09	0.08	0.69	0.32–1.50	0.35
	50–69 yrs	0.69	0.34–1.39	0.29	0.96	0.44–2.05	0.91
BMI (reference = not overweight)^a^	Overweight or obese	0.59	0.036–0.96	0.03	0.54	0.32–0.92	0.02
Home ownership (reference = rents)	Owns	0.82	0.47–1.43	0.49	-	-	-
Number of cars (reference = none)^a^	1	0.57	0.27–1.24	0.15	0.51	0.23–1.15	0.11
2 or more cars		0.58	0.27–1.23	0.15	0.50	0.22–1.15	0.10
Highest educational qualification (reference = up to degree level)^a^	Degree level or higher	0.63	0.38–1.02	0.06	0.68	0.41–1.13	0.13
Travel to work by bicycle (reference = never)^a^	Occasionally, usually or always	0.69	0.44–1.08	0.11	0.64	0.39–1.04	0.07
**PA ranked higher by self-report than accelerometer (n = 163)**							
Sex (reference = female)^a^	Male	1.65	1.04–2.61	0.04	1.58	0.97–2.58	0.07
Age (reference = 20–29 yrs)^a^	30–39 yrs	0.91	0.42–1.95	0.80	0.79	0.36–1.75	0.56
40–49 yrs		1.10	0.47–2.17	0.97	0.78	0.35–1.78	0.56
50–69 yrs		1.07	0.50–2.26	0.87	0.75	0.33–1.71	0.50
BMI (reference = not overweight)^a^	Overweight or obese	2.02	1.29–3.17	0.002	2.07	1.28–3.34	0.003
Home ownership (reference = rents)	Owns	0.94	0.54–1.64	0.81	-	-	-
Number of cars (reference = none)^a^	1	0.93	0.41–2.13	0.86	1.24	0.51–3.02	0.64
2 or more cars		1.01	0.45–2.29	0.98	1.10	0.46–2.65	0.83
Highest educational qualification (reference = up to degree level)^a^	Degree level or higher	0.85	0.52–1.41	0.53	0.80	0.48–1.36	0.41
Travel to work by bicycle (reference = never)^a^	Occasionally, usually or always	1.26	0.81–1.95	0.31	1.37	0.85–2.22	0.20

OR = odds ratio. Data were collected between May and November 2009 in Cambridge, UK.

a p<0.25 in univariate analysis therefore variable carried forward into multivariate analysis.

Among men, individuals whose PA was ranked higher by accelerometer than by self-report were more likely to be of normal weight and living in a household without a car (both p<0.25) (Table 5). These variables were therefore carried forward into the multivariable analysis for men.

**Table 5:** Associations between individual characteristics and the ranking of physical activity by self-report and an accelerometer in males.

		*Univariate Analysis*			*Multivariable Analysis*		
		OR	95% CI	p	OR	95% CI	p
**PA ranked higher by accelerometer than self-report (n = 29)**							
Age (reference = 20–29 yrs)	30–39 yrs	0.85	0.25–7.08	0.74	-	-	-
40–49 yrs		0.37	0.06–2.23	0.28	-	-	-
50–69 yrs		1.33	0.14–4.99	0.86	-	-	-
BMI (reference = not overweight)^a^	Overweight or obese	0.46	0.18–1.21	0.12	0.50	0.19–1.31	0.16
Home ownership (reference = rents)	Owns	0.91	0.29–2.88	0.87	-	-	-
Number of cars (reference = none)^a^	1	0.34	0.09–1.31	0.07	0.36	0.09–1.39	0.14
2 or more cars		0.28	0.07–1.09	0.12	0.31	0.08–1.20	0.09
Highest educational qualification (reference = up to degree level)	Degree level or higher	0.91	0.23–3.56	0.9	0.88	0.22–3.53	0.86
Travel to work by bicycle (reference = never)	Occasionally, usually or always	0.99	0.39–2.52	0.99	-	-	-
**PA ranked higher by self-report than accelerometer (n = 68)**							
Age (reference = 20–29 yrs)	30–39 yrs	1.81	0.40–8.73	0.46	-	-	-
40–49 yrs		1.39	0.31–6.30	0.67	-	-	-
50–69 yrs		1.36	0.29–6.38	0.7	-	-	-
BMI (reference = not overweight)	Overweight or obese	0.74	0.35–1.55	0.42	0.70	0.33–1.51	0.37
Home ownership (reference = rents)	Owns	1.11	0.42–2.88	0.84	-	-	-
Number of cars (reference = none)	1	0.82	0.23–3.00	0.77	0.79	0.21–2.95	0.73
2 or more cars		1.23	0.35–4.36	0.74	1.28	0.36–4.61	0.70
Highest educational qualification (reference = up to degree level)^a^	Degree level or higher	0.44	0.16–1.21	0.11	0.42	0.15–1.16	0.10
Travel to work by bicycle (reference = never)	Occasionally, usually or always	1.39	0.65–2.96	0.4	-	-	-

OR = odds ratio. Data were collected between May and November 2009 in Cambridge, UK.

a p<0.25 therefore variable carried forward into multivariate analysis.

Among women, individuals whose PA was ranked higher by accelerometer than by self-report were less likely to be older, to be educated to degree level or to cycle to work (p<0.25) (Table 6). A higher BMI was associated with PA being ranked higher by self-report than by accelerometer (p<0.25). These variables were therefore carried forward into the multivariable analysis for women.

**Table 6:** Associations between individual characteristics and the ranking of physical activity by self-report and an accelerometer in females.

		*Univariate Analysis*			*Multivariable Analysis*		
		OR	95% CI	p	OR	95% CI	p
**PA ranked higher by accelerometer than self-report (n = 131)**							
Age (reference = 20–29 yrs)^a^	30–39 yrs	0.54	0.24–1.20	0.13	0.53	0.23–1.20	0.13
40–49 yrs		0.66	0.30–1.52	0.33	0.64	0.27–1.50	0.30
50–69 yrs		0.62	0.28–1.37	0.24	0.61	0.26–1.40	0.25
BMI (reference = not overweight)	Overweight or obese	0.74	0.41–1.32	0.31	0.68	0.37–1.27	0.23
Home ownership (reference = rents)	Owns	0.80	0.42–1.51	0.49	-	-	-
Number of cars (reference = none)	1	0.66	0.25–1.72	0.40	-	-	-
2 or more cars		0.68	0.26–1.73	0.41	-	-	-
Highest educational qualification (reference = up to degree level)^a^	Degree level or higher	0.67	0.39–1.15	0.15	0.67	0.39–1.17	0.16
Travel to work by bicycle (reference = never)^a^	Occasionally, usually or always	0.69	0.40–1.15	0.15	0.63	0.36–1.08	0.09
**PA ranked higher by self-report than accelerometer (n = 95)**							
Age (reference = 20–29 yrs)	30–39 yrs	0.63	0.26–1.55	0.93	0.52	0.20–1.36	0.18
40–49 yrs		0.79	0.31–1.99	0.61	0.69	0.26–1.85	0.47
50–69 yrs		0.96	0.40–2.29	0.93	0.61	0.23–1.57	0.30
BMI (reference = not overweight)^a^	Overweight or obese	3.57	1.99–6.39	0.0001	3.97	2.11–7.47	0.0001
Home ownership (reference = rents)	Owns	0.84	0.42–1.68	0.62	-	-	-
Number of cars (reference = none)	1	1.07	0.36–3.20	0.91	-	-	-
2 or more cars		0.96	0.32–2.84	0.94			
Highest educational qualification (reference = up to degree level)	Degree level or higher	1.01	0.55–1.84	0.99	1.13	0.60–2.13	0.72
Travel to work by bicycle (reference = never)	Occasionally, usually or always	1.07	0.62–1.87	0.80	1.29	0.70–2.34	0.41

OR = odds ratio. Data were collected between May and November 2009 in Cambridge, UK.

a p<0.25 therefore variable carried forward into multivariate analysis.

### Multivariable Analyses

In adjusted analyses in the full sample, the PA of overweight or obese individuals was significantly less likely to be ranked in a higher quartile by an accelerometer than that of normal weight individuals (OR = 0.54, 95%CI = 0.32–0.92) and twice as likely as that of normal weight individuals to be ranked in a higher quartile by self-report (OR = 2.07, 95%CI = 1.28–3.34) (Table 4). In analysis stratified by sex, the PA of overweight or obese women was almost four times more likely than that of normal weight women to be ranked in a higher quartile by self-report than by accelerometer (OR = 3.97, 95%CI = 2.11–7.47) (Table 6).

## Discussion

The results of this study demonstrate that, when considered individually, characteristics such as sex, age, education, car access, cycling to work and BMI were at least weakly associated with differences in the ranking of PA by self-report and accelerometry. When subsequently considered together in multivariable analyses, BMI was the only factor that remained significant.

Our finding that a BMI greater than 25 kg/m^2^ was associated with PA being ranked higher by self-report than by accelerometer in the full cohort and in women is in keeping with the findings of Walsh et al [14], who demonstrated that overweight American black and white women overestimated their total EE 49% more than control subjects who had never been overweight. We have demonstrated that the PA of overweight or obese women was four times more likely than that of normal-weight women to be ranked higher by self-report than by accelerometer. This finding was not observed for men, despite the fact that a similar proportion of overweight or obese men and women were categorised as having been ranked higher by self-report than by accelerometer (47% vs. 47.9%). Further study is required to clarify whether this reflects a true difference between the sexes or simply the smaller sample of men available for analysis in this study.

Differences associated with BMI in the ranking of PA between the two measurement techniques may result from a number of factors. Firstly, overweight or obese individuals may be more likely to present themselves in a positive light according to their perceived cultural norms. This social desirability bias has previously been shown to be related to the over-reporting of self-reported PA [26]. Adams et al. did not find a relationship between BMI and social desirability bias in their study, but a subsequent study [11] has shown that individuals who, rightly or not, consider their weight to be appropriate more often assume that their PA is adequate or high, because PA is often proposed as a method of weight loss. Misperceptions about weight status amongst overweight or obese females may therefore also lead to overestimation of activity levels, although further research is required to confirm the direction of any such effect.

A second consideration is that the accelerometer may underestimate actual PA because it cannot be relied upon to record cycling or aquatic activities adequately [27]. In this study, 43% of overweight or obese adults reported spending any time cycling to and from work [16] and cycling for recreation in the last four weeks was also relatively common in the sample (69% of men and 56% of women reported doing so) [25]. Although we found that those who cycled to work at least ‘occasionally’ were less likely to be ranked higher by accelerometer than by self-report and more likely to be ranked higher by self-report than by accelerometer in multivariable models, these results were not statistically significant (p = 0.07 and p = 0.20 respectively). The categorical measures of cycling used here do not capture the quantity of cycling on the journey to and from work, but the direction of these associations suggests considerable potential for under-ascertainment of activity by accelerometry in some individuals.

### Strengths and Limitations

In this study we have not analysed the correlates of mismatches of measurement techniques based on absolute differences in PA. Though in some circumstances this might be desirable, a recent discussion article [9] highlighted the need to consider data from self-report and accelerometry as qualitatively different. Haskell argues that neither should be considered to estimate an absolute quantity of PA, as accelerometers detect features of PA that are not captured by self-report and vice versa. We therefore chose to use both instruments to rank individual’s PA to assess the correlates of any mismatches in either direction.

A number of further limitations should be noted in the interpretation of the findings. Firstly, the sample was composed of employed adults, mostly well-educated and economically relatively advantaged and was therefore not representative of the general population. Further research is required to understand the impact of factors linked to lower educational attainment, such as low literacy levels on self-reported PA levels [28]. Our sample also contained relatively few men. The analysis was stratified by sex because of an interaction between sex and BMI, but this resulted in a group of only 144 males which it was not appropriate to divide further (for example by age group) for more detailed group comparisons. Further research in a larger sample is required to confirm the findings of this study.

## Conclusions

With the exception of BMI, no individual characteristics were associated with mismatches between self-reported and accelerometer-measured PA in this study. Given that data from self-report and accelerometry may be considered as qualitatively different, and that the PA of overweight or obese adults was more likely to be ranked higher using self-reported methods, future research should explore methods of aligning these complementary approaches to physical activity measurement. For example, statistical approaches such as structural equation modelling permit a complex phenomenon such as ‘physical activity’ to be modelled as a latent construct imperfectly represented by a number of complementary, directly observed variables [29], [30]. Quantifying the differences between the two measurement techniques and investigating ways of meaningfully combining them would appear logical first steps towards a more integrated approach to the analysis of physical activity.
